# Impact of Coronavirus Disease 2019 Pandemic on Pediatric Out-of-Hospital Cardiac Arrest in the Emergency Department

**DOI:** 10.3389/fped.2022.846410

**Published:** 2022-04-25

**Authors:** Chun-Yu Chen, En-Pei Lee, Yu-Jun Chang, Wen-Chieh Yang, Mao-Jen Lin, Han-Ping Wu

**Affiliations:** ^1^Department of Pediatric Emergency, China Medical University Children’s Hospital, China Medical University, Taichung City, Taiwan; ^2^Department of Medicine, College of Medicine, China Medical University, Taichung City, Taiwan; ^3^Division of Pediatric Critical Care Medicine, Department of Pediatrics, Chang Gung Memorial Hospital at Linko, Taoyuan, Taiwan; ^4^College of Medicine, Chang Gung University, Taoyuan, Taiwan; ^5^Laboratory of Epidemiology and Biostastics, Changhua Christian Hospital, Changhua City, Taiwan; ^6^Department of Medicine, College of Medicine, Tzu Chi University, Hualien City, Taiwan; ^7^Division of Cardiology, Department of Medicine, Taichung Tzu Chi Hospital, The Buddhist Tzu Chi Medical Foundation, Taichung City, Taiwan; ^8^Department of Medical Research, China Medical University Children’s Hospital, China Medical University, Taichung City, Taiwan

**Keywords:** OHCA, out-of-hospital cardiac arrest, COVID-19, emergency department, children

## Abstract

**Background:**

Out-of-hospital cardiac arrest (OHCA) in children is a critical condition with a poor prognosis. After the coronavirus disease 2019 (COVID-19) pandemic developed, the epidemiology and clinical characteristics of the pediatric emergency department (PED) visits have changed. This study aimed to analyze the impact of the COVID-19 pandemic on pediatric OHCA in the PED.

**Methods:**

From January 2018 to September 2021, we retrospectively collected data of children (18 years or younger) with a definite diagnosis of OHCA admitted to the PED. Patient data studied included demographics, pre-/in-hospital information, treatment modalities; and outcomes of interest included sustained return of spontaneous circulation (SROSC) and survival to hospital-discharge (STHD). These were analyzed and compared between the periods before and after the COVID-19 pandemic.

**Results:**

A total of 97 patients with OHCA (68 boys and 29 girls) sent to the PED were enrolled in our study. Sixty cases (61.9%) occurred in the pre-pandemic period and 37 during the pandemic. The most common age group was infants (40.2%) (*p* = 0.018). Asystole was the most predominant cardiac rhythm (72.2%, *P* = 0.048). Eighty patients (82.5%) were transferred by the emergency medical services, 62 (63.9%) gained SROSC, and 25 (25.8%) were STHD. During the COVID-19 pandemic, children with non-trauma OHCA had significantly shorter survival duration and prolonged EMS scene intervals (both *p* < 0.05).

**Conclusion:**

During the COVID-19 pandemic, children with OHCA had a significantly lower rate of SROSC and STHD than that in the pre-pandemic period. The COVID-19 pandemic has changed the nature of PED visits and has affected factors related to ROSC and STHD in pediatric OHCA.

## Introduction

Pediatric out-of-hospital cardiac arrest (OHCA) is a rare but critical condition with a very poor outcome in the emergency department (ED) (1). The major causes of OHCA in childhood are of non-cardiac etiology, including respiratory failure, drowning, accident, child abuse, and airway obstruction. The survival rate of pediatric patients with OHCA remains low (6.4–8%) (2, 3). Previous studies have identified the factors associated with improving survival, including bystander cardiopulmonary resuscitation (CPR), witnessed OHCA, initial shockable rhythm, chest compression-only CPR, shorter duration of transportation, and early epinephrine administration (4–11).

Severe acute respiratory syndrome coronavirus 2 (SARS-CoV-2) caused a global pandemic, and the World Health Organization declared the outbreak a pandemic on 11 March 2020 (12–14). On 2 January 2020, the Taiwan Centers for Disease Control (Taiwan CDC) response team was set up and it classified the coronavirus disease 2019 (COVID-19) as a Category 5 communicable disease on 15 January 2020. The impact of the COVID-19 pandemic on the ED use in the United States had decreased by 42% after more than 1 month following school closure (15). On examining the 4-month period following school closure, a previous study covering 210 hospitals in Japan found reductions in pediatric hospitalization for infectious disease ranging from 41% at the end of March to 74% at the end of May 2020 (16). In Taiwan, we have established epidemic prevention regulations, including face masks, adequate social distancing, hand washing, and alcohol-based disinfectant. However, schools and businesses were not closed. The detailed nature of the pediatric OHCA changes during the COVID-19 pandemic has not been analyzed adequately. This study aimed to analyze the epidemiology and clinical spectrum of OHCA in children admitted to the pediatric ED (PED) before and after the COVID-19 pandemic outbreak and survey the impact of the pandemic in pediatric OHCA admitting hospital characteristics and outcome.

## Materials and Methods

This multicenter study retrospectively collected data of all pediatric patients with a discharge diagnosis of OHCA aged 18 years or younger sent to the EDs in China Medical University Children’s Hospital, Central Taiwan; and Chang Gung Memorial Hospital, Northern Taiwan. We reviewed the medical charts of all eligible patients from January 2018 to September 2021. Pediatric cardiac arrest (CA) was defined as unresponsiveness with an absent palpable pulse, no signs of life, or healthcare provider perceived need for chest compressions for at least 1 min. We divided these patients into two groups based on the admission period: Group 1 (pre-pandemic period, January 2018 to December 2019) and Group 2 (pandemic period, January 2020 to September 2021). We excluded the patients who: (1) were aged >18 years, (2) had not presented to the ED before the admission, and (3) had “do not resuscitate” orders. A total of 97 patients were included in the study. The study was approved by the Institutional Review Board of the China Medical University Hospital (CMUH110-REC3-083). All procedures were performed in accordance with the relevant guidelines and regulations (17). Data were collected, reviewed, de-identified, and anonymized before analysis, and the Ethics Committee waived the requirement for informed consent because of the anonymized nature of the data and the scientific purpose of the study.

The following information was obtained from the medical records of each patient: basic demographic characteristics (i.e., age, sex, and pre-existing medical conditions), category of etiology, comorbidities, timing, initial heart rhythm, first blood lactate and pH on hospital arrival, place, and short-term outcomes. In addition, pre-ED information was obtained from the emergency medical services (EMS) records, including the time the call was received, the time of arrival and departure from the scene, the time the patient arrived at the ED, pre-hospital defibrillation by AED, and the duration of pre-ED CPR. All data were collected from medical records and analyzed retrospectively. The duration of ED CPR was defined as the time interval from the time CPR was initiated to the time it was stopped at the ED. Sustained return of spontaneous circulation (SROSC) was defined as the restoration of perfusion and heart rhythm in the absence of external chest compressions for over 20 min (18). Furthermore, patients were divided into four age groups: infants (≤1 year), young children (2−6 years), older children (7−12 years), and adolescents (13−18 years).

### Statistical Analysis

Data of categorical variables were analyzed using the Chi-square test or Fisher’s exact test, when appropriate. Continuous variables were analyzed by the Mann–Whitney *U*-Test and the Jonckheere–Terpstra test. A *p*-value less than 0.05 was considered to be statistically significant. Distributions of variables were reported as percentages and means ± standard deviation (SD). We also performed an analysis after adjusting for confounding factors of the risk of mortality (Cox regression analysis). In addition, a Kaplan–Meier survival analysis was performed according to the period of admission in all patients who achieved survival to hospital discharge (STHD). Statistical analyses were performed with SPSS software (version 15.0, SPSS, Inc., Chicago, IL, United States).

## Results

### Demographics of Children With Out-of-Hospital Cardiac Arrest

Between January 2018 and September 2021, about 212,100 pediatric ED visits were recorded, of which 97 pediatric patients (68 males and 29 females) who presented to the ED with OHCA were enrolled. Thus, the incidence of OHCA in children admitted to the pediatric ED was 0.046%. The incidence of OHCA in children was 2.5 per month before the pandemic, which dropped to 1.77 per month during the pandemic. Demographics and resuscitation information are shown in Table 1. Of all patients, 61.9% (*n* = 60) were in Group 1 and most were infants (*n* = 39, 40.2%, *p* = 0.018). Asystole was the most predominant non-shockable cardiac rhythm in both the groups (*n* = 70, 72.2%, *P* = 0.048). OHCA was witnessed in 41 (42.3%) children and bystander CPR was delivered in 56 (57.7%) cases. The EMS transferred 80 patients (82.5%), and 67 (69.1%, *P* = 0.039) were initially resuscitated by pediatricians in the ED. Overall, 63.9% (*n* = 62) of cases gained SROSC and 25.8% (*n* = 25) were STHD.

**TABLE 1:** Demographics and associations with outcomes of the patients with OHCA.

		Group 1 (*n* = 60)		Group 2 (*n* = 37)		*P*-value
		*N*	%	*N*	%	
Age	< = 1	26	43.3	13	35.1	0.018*
	2–6	21	35.0	7	18.9	
	7–12	2	3.3	8	21.6	
	13–18	11	18.3	9	24.3	
Gender	Male	45	75.0	23	62.2	0.180
	Female	15	25.0	14	37.8	
Pre-existing conditions	Respiratory	3	5.0	1	2.7	0.106
	Cardiac	1	1.7	0	0.0	
	Neurologic	6	10.0	0	0.0	
	Gastrointestinal	2	3.3	1	2.7	
	Prenatal	1	1.7	3	8.1	
	Other conditions	9	15.0	11	29.7	
	None	38	63.3	21	56.8	
Initial arrest rhythm	Asystole	43	71.7	27	73.0	0.048*
	Pulseless electrical activity (PEA)	8	13.3	9	24.3	
	Ventricular fibrillation (VF)	0	0.0	1	2.7	
	Pulseless ventricular tachycardia (pVT)	3	5.0	0	0.0	
	Unknown	6	10.0	0	0.0	
Witnessed	No	36	60.0	20	54.1	0.565
	Yes	24	40.0	17	45.9	
Bystander compressions performed	No	27	45.0	14	37.8	0.488
	Yes	33	55.0	23	62.2	
Prompt access to the emergency response system	Sent by EMS	49	81.7	31	83.8	0.790
	Sent by caretaker	11	18.3	6	16.2	
Rapid pediatric advanced life support (PALS)	Pediatrician	46	76.7	21	56.8	0.039*
	Others	14	23.3	16	43.2	
Shift	Day shift (7A.M. to 7P.M.)	35	58.3	20	54.1	0.679
	Night shift (7P.M. to 7A.M)	25	41.7	17	45.9	

*Statistically significant by theχ2 test or Fisher’s exact test when appropriated. Age, years.

### Causes of Pediatric Out-of-Hospital Cardiac Arrest

The four most common causes of pediatric OHCA before the pandemic were unknown medical causes (25%, *n* = 25), sudden infant death syndrome (SIDS) (16.7%, *n* = 10), choking (15%, *n* = 9), and trauma (13.4%, *n* = 8). During the pandemic, most common causes of pediatric OHCA showed some change: unknown medical cause (37.8%, *n* = 14), trauma (13.5%, *n* = 5), SIDS (10.8%, *n* = 4), and precipitate delivery (10.8%, *n* = 4) (Table 2).

**TABLE 2:** Manner of pediatric OHCA.

		Group 1 (*n* = 60)		Group 2 (*n* = 37)		*P*-value
		*N*	%	*N*	%	
Etiologies of pediatric OHCA	Unknown	15	25.0	14	37.8	0.161
	Sudden infant death syndrome (SIDS)	10	16.7	4	10.8	
	Trauma (Traffic accident)	3	5.0	3	8.1	
	Trauma (Fall)	4	6.7	2	5.4	
	Trauma (Other injury)	1	1.7	0	0.0	
	Choking	9	15.0	2	5.4	
	Suspected child abuse	7	11.7	1	2.7	
	Precipitate delivery	0	0.0	4	10.8	
	Drowning	5	8.3	3	8.1	
	Epilepsy	4	6.7	2	5.4	
	House fire	1	1.7	2	5.4	
	Suicide	1	1.7	0	0.0	

*P-value by Chi-Square Test or Fisher’s exact test when appropriated.*

### Prompt Access to the Emergency Medical Services and Rapid Pediatric Advanced Life Support

The EMS transferred 80 patients (82.5%), and all received life support with oxygenation and chest compressions. During the pandemic, the median time of EMS transport became longer (group 1: 22.5 min vs. group 2: 33.5 min), and the EMS scene interval of non-trauma patients in group 2 was significantly longer than that of group 1 (15.7 ± 8.9 min vs. 10.3 ± 8.9 min, respectively; *p* = 0.019, not shown; Table 3). The duration of chest compressions after hospital arrival was 30.4 ± 32.2 min in group 1 and 26.1 ± 18 min in group 2. The first epinephrine time after hospital arrival was 3 ± 3.4 min in group 1 and 2.7 ± 3.4 min in group 2.

**TABLE 3:** Comparison of rapid pediatric advanced life support (PALS) and time frame of emergency response system (min) based on before or after the pandemic.

	Study group	*N*	Mean	SD	Median	IQR	*P*-value
Age	Group 1	60	4.2	5.6	1.3	3.9	0.293
	Group 2	37	6.5	6.4	5.3	9.4	
Duration of chest compressions after hospital	Group 1	56	30.4	32.2	30.0	22.5	0.806
	Group 2	34	26.1	18.0	29.0	26.0	
First epinephrine time (min) at ED	Group 1	44	3.0	3.4	2.0	3.5	0.574
	Group 2	25	2.7	3.4	3.0	4.0	
Number of doses of epinephrine administered during the arrest	Group 1	49	6.7	6.0	6.0	8.0	0.771
	Group 2	32	6.3	5.9	4.5	10.0	
Duration of chest compressions before hospital	Group 1	44	7.2	6.9	5.0	12.0	0.715
	Group 2	26	7.7	6.2	9.5	13.0	
PH	Group 1	50	7.0	0.3	7.0	0.4	0.801
	Group 2	32	7.0	0.3	7.0	0.4	
Glucose	Group 1	43	264.6	129.7	280.0	201.0	0.209
	Group 2	29	232.3	164.1	225.0	270.0	
Lactate (mg/dL)	Group 1	40	108.3	60.8	112.7	87.4	0.769
	Group 2	22	105.5	69.1	100.4	116.3	
EMS response interval (min)	Group 1	39	9.1	7.2	6.0	11.0	0.088
	Group 2	24	11.9	7.5	10.0	13.0	
EMS scene interval (min)	Group 1	38	12.0	8.6	9.0	13.0	0.065
	Group 2	24	16.0	8.9	14.5	16.0	
EMS scene to ED interval (min)	Group 1	38	15.2	27.1	7.5	10.0	0.329
	Group 2	23	14.3	11.2	9.0	14.0	

*Statistically significant by Mann–Whitney U-Test. IQR, interquartile range.*

### Comparison of Child Characteristics and Associations With Outcomes Based on the Pandemic Period

Most patients were infants (*n* = 39, 40.2%), followed by toddlers and preschool-age children (*n* = 29, 28.5%; Table 4). We found an increasing trend in the age distribution of pediatric OHCA during the pandemic: from 21.6 to 45.9% in the age group of 7–18 years. The overall mortality rate of group 2 was higher than that of group 1 (86.5 vs. 66.7%). Asystole was the predominant cardiac rhythm in all pediatric patients with OHCA (*n* = 70, 72.2%, *p* = 0.023) and was the predominant rhythm in both survival and non-survival sub-groups in groups 1 and 2. Before the pandemic, the initial cardiac rhythm in pediatric patients with OHCA showed asystole with a higher mortality rate than other initial cardiac rhythms (*P* = 0.004), and patients sent to the ER during the day shift had a higher survival rate than in the night shift (*p* < 0.001). Moreover, we found that witnessed OHCA, bystander CPR, rapid PALS by a pediatrician, OHCA caused by trauma, EMS transport, and EMS advanced airway management were not associated with the outcomes of interest.

**TABLE 4:** Comparison of child characteristics and associations with outcomes based on before or after pandemic.

		Group 1							Group 2						
		Mortality							Mortality						
		No (*n* = 20)		Yes (*n* = 40)		Total (*n* = 60)		*P*-value	No (*n* = 5)		Yes (*n* = 32)		Total (*n* = 37)		*P*-value
		N	%	N	%	N	%		N	%	N	%	N	%	
Age (years)	< = 1	8	40.0	18	45.0	26	43.3	0.664	1	20.0	12	37.5	13	35.1	0.850
	2–6	9	45.0	12	30.0	21	35		1	20.0	6	18.8	7	18.9	
	7–12	0	0.0	2	5.0	2	3.3		2	40.0	6	18.8	8	21.6	
	13-18	3	15.0	8	20.0	11	18.3		1	20.0	8	25.0	9	24.3	
Gender	Male	14	70.0	31	77.5	45	75.0	0.527	3	60.0	20	62.5	23	62.2	1.000
	Female	6	30.0	9	22.5	15	25.0		2	40.0	12	37.5	14	37.8	
Pre-existing conditions	Respiratory	3	15.0	0	0.0	3	5.0	0.01	0	0.0	1	3.1	1	2.7	0.58
	Cardiac	0	0.0	1	2.5	1	1.7		0	0.0	0	0.0	0	0.0	
	Neurologic	0	0.0	6	15.0	6	10.0		0	0.0	0	0.0	0	0.0	
	Gastrointestinal	2	10.0	0	0.0	2	3.3		0	0.0	1	3.1	1	2.7	
	Prenatal	0	0.0	1	2.5	1	1.7		0	0.0	3	9.4	3	8.1	
	Other conditions	4	20.0	5	12.5	9	15.0		3	60.0	8	25.0	11	29.7	
	None	11	55.0	27	67.5	38	63.3		2	40.0	19	59.4	21	56.8	
Initial arrest rhythm	Asystole	10	50.0	33	82.5	43	71.7	0.004	4	80.0	23	71.9	27	73.0	0.083
	PEA	6	30.0	2	5.0	8	13.3		0	0.0	9	28.1	9	24.3	
	VF	0	0.0	0	0.0	0	0.0		1	20.0	0	0.0	1	2.7	
	pVT	0	0.0	3	7.5	3	5.0		0	0.0	0	0.0	0	0.0	
	Unknown	4	20.0	2	5.0	6	10.0		0	0.0	0	0.0	0	0.0	
Witnessed	Yes	11	55.0	13	32.5	24	40.0	0.094	4	80.0	13	40.6	17	45.9	0.159
Bystander compressions	Yes	12	60.0	21	52.5	33	55.0	0.582	2	40.0	21	65.6	23	62.2	0.346
Transfer	Sent by EMS	18	90.0	31	77.5	49	81.7	0.307	5	100.0	26	81.3	31	83.8	0.567
	Sent by caretaker	2	10.0	9	22.5	11	18.3		0	0.0	6	18.8	6	16.2	
EMS advanced airway	Yes	1	5.0	1	2.5	2	3.3	1.000	0	0.0	1	3.1	1	2.7	1.000
Rapid PALS	Pediatrician	15	75.0	31	77.5	46	76.7	1.000	3	60.0	18	56.3	21	56.8	1.000
	Others	5	25.0	9	22.5	14	23.3		2	40.0	14	43.8	16	43.2	
Shift	Day shift (7A.M. to 7P.M.)	18	90.0	17	42.5	35	58.3	<0.001	3	60.0	17	53.1	20	54.1	1.000
	Night shift (7P.M. to 7A.M)	2	10.0	23	57.5	25	41.7		2	40.0	15	46.9	17	45.9	
STHD	Yes	20	100.0	0	0.0	20	33.3	<0.001	5	100.0	0	0.0	5	13.5	<0.001
SROSC	Yes	20	100.0	22	55.0	42	70	<0.001	5	100.0	15	46.9	20	54.1	0.05
Trauma	Yes	9	45.0	13	32.5	22	36.7	0.344	1	20.0	10	31.3	11	29.7	1.000
Manner of pediatric OHCA	Unknown	2	10.0	13	32.5	15	25.0	0.129	3	60.0	11	34.4	14	37.8	0.792
	SIDS	2	10.0	8	20.0	10	16.7		0	0.0	4	12.5	4	10.8	
	Trauma (Traffic accident)	1	5.0	2	5.0	3	5.0		0	0.0	3	9.4	3	8.1	
	Trauma (Fall)	1	5.0	3	7.5	4	6.7		0	0.0	2	6.3	2	5.4	
	Trauma (Other injury)	0	0.0	1	2.5	1	1.7		0	0.0	0	0.0	0	0.0	
	Choking	4	20.0	5	12.5	9	15.0		0	0.0	2	6.3	2	5.4	
	Suspected child abuse	3	15.0	4	10.0	7	11.7		0	0.0	1	3.1	1	2.7	
	Precipitate delivery	0	0.0	0	0.0	0	0.0		0	0.0	4	12.5	4	10.8	
	Drowning	4	20.0	1	2.5	5	8.3		1	20.0	2	6.3	3	8.1	
	Epilepsy	3	15.0	1	2.5	4	6.7		1	20.0	1	3.1	2	5.4	
	House fire	0	0.0	1	2.5	1	1.7		0	0.0	2	6.3	2	5.4	
	Suicide	0	0.0	1	2.5	1	1.7		0	0.0	0	0.0	0	0.0	

*P-value by Chi-Square Test or Fisher’s exact test when appropriated. PEA, pulseless electrical activity; VF, ventricular fibrillation; pVT, pulseless ventricular tachycardia; EMS, emergency medical service; PALS, pediatric advanced life support; STHD, survival to hospital discharge; SROSC, sustained return of spontaneous circulation; SIDS, sudden infant death syndrome.*

### Survival Curves for Different Periods of Pandemic

**FIGURE 1:**
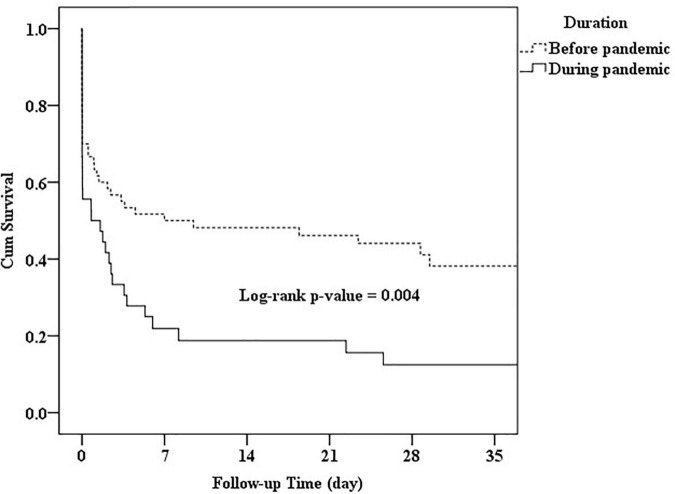
Survival duration of pediatrics with out-of-hospital cardiac arrest (OHCA) according to the period before or during the pandemic.

The associations between the survival duration and periods before and during the pandemic are shown in Figure 1. Patients who presented with OHCA before the pandemic had a longer survival duration than those who presented during the pandemic (*p* = 0.004).

## Discussion

Pediatric OHCA is a relatively rare, but one of the most critical conditions presenting to the ED. In our study, the incidence of OHCA in children admitted to the pediatric ED was 46 of every 100,000 pediatric ED visits. The incidence of pediatric OHCA has been reported to be 8.0–23 per 100,000 persons per year (2, 19, 20). During our study period, the most common age was infancy, and the incidence was higher in males with a male to female ratio of 2.34:1. This result is similar to that of previous studies, but our study results showed a higher male-to-female ratio (18–21). Medically fragile children (with pre-existing conditions: overall: 39.2%; group 1: 37.7%; group 2: 43.2%) were at a high risk for OHCA, both before and during the pandemic. Asystole was the most predominant non-shockable cardiac rhythm before and during the pandemic. OHCA was witnessed in 41 children (group 1: *n* = 24, 40%; group 2: *n* = 17, 45.9%), and bystander CPR was delivered in 56 cases (group 1: *n* = 33, 55%; group 2: *n* = 23, 62.2%). The EMS transported 80 patients (82.5%), and this was similar to a previous study (81%) (2). However, resuscitation rate by pediatricians in the ED was significantly reduced during the pandemic (group 1: *n* = 46, 76.7%; group 2: *n* = 21, 56.8%; *P* = 0.039).

Before the pandemic, unknown medical causes, SIDS, and choking were the three most common causes of pediatric OHCA. During the pandemic, unknown medical causes, trauma, SIDS, and precipitate delivery were the four most common causes. Unknown medical causes were the most common OHCA trigger both before and during the pandemic, and it meant that etiological diagnosis was difficult; only the clinical information and autopsy could clarify the exact cause of death. In addition, we found that pediatric OHCA caused by choking and child abuse decreased by approximately 10%, and precipitate delivery increased by approximately 10% during the pandemic. None of the children who presented with OHCA had COVID-19 in our study.

As for EMS response time, multiple studies have shown good OHCA outcomes with shorter EMS response times. However, Cheng et al. showed that the EMS response time was related to higher odds of STHD for adults, but the difference was not statistically significant for pediatric patients (22). The present study showed similar results where EMS response interval, EMS scene interval, and EMS scene to ED interval were not statistically significant factors for STHD of pediatric OHCA in both the before and during pandemic groups. In addition, we found that the EMS scene interval of non-trauma patients in the pandemic period group was significantly longer than that of the before pandemic period group. However, we could not elucidate the major cause of this based on our limited documentation.

In previous studies, regardless of whether the patients were adults or pediatric patients, OHCA that occurs at night has been associated with a poor survival rate (23–27). In the before pandemic group, we found that daytime admission had significantly more survivors than non-survivors (58.3 vs. 41.7%; *p* < 0.01, respectively). However, during the pandemic group, results showed no significant relationship between daytime admission and STHD. In addition, the overall survival rate of OHCA occurring at day was significantly higher than that occurring at night (38.2% vs. 9.5%; *p* < 0.01, respectively). Further studies are required to clarify this issue. In our study, the SROSC rate of all children was 63.9% (group 1: 70%; group 2: 54.1%), higher than that in other published reports where it was between 27.8 and 34.9% (18–20). This may be explained by the fact that we did not exclude patients (*n* = 21, 21.6%) transferred from another ED after ROSC. The STHD rate was 25.8% (group 1: 33.3%; group 2: 13.5%), within the range of results from earlier studies (8.2–26.9%) (3, 18–21, 28). We found that the pediatric OHCA frequency, the SROSC rate, and the STHD rate were reduced during the pandemic.

After the COVID-19 pandemic, enhanced public health measures were enacted, particularly hand hygiene, physical distancing, face masks, restrictions to travel, and social gatherings. However, school and business closures were not implemented. Based on the previous studies, we found that school closure from 1 to 3 months may lead to a decline of ∼60% in pediatric ED volume (29, 30). The COVID-19 pandemic reduced our pediatric ED visits by more than 40%. This may indicate that significant changes have developed in ED patient demographics and visit characteristics. Further study is required to distinguish the changes in disease patterns and pediatric OHCA from health-seeking behaviors and established epidemic prevention regulations. The causes and outcomes of pediatric OHCA before and during the pandemic differ. We found that patients who presented with OHCA before the pandemic had longer survival durations than those during the pandemic (*p* = 0.004). Before the pandemic, 13 of 60 pediatric OHCA children were caused by trauma (*n* = 8) and drowning (*n* = 5), and 5 of these 13 patients, aged younger than 6 years, achieved STHD. During the pandemic, 12 of 37 pediatric OHCA children were caused by trauma (*n* = 5), precipitate delivery (*n* = 4), and drowning (*n* = 3), and only one 10-year-old boy achieved STHD. We believe that the etiology and the age of pediatric patients with OHCA may play an important role in survival duration and STHD. In addition, some of the predictors of improved survival seem to have been changed by the COVID-19 pandemic. Further study is needed to determine the relationship between STHD and the severity of the COVID-19 pandemic.

### Limitations

The present study has some limitations. First, this was a retrospective study, and the data or timing might not have been well-documented due to the rush during OHCA events at the scene or in the ED. Second, the data was collected by two different hospitals, and we did not exclude the pediatric patients with OHCA transferred from other hospitals, which may have different medical facilities and the transfer distance to our hospital may also vary. Third, we were unable to measure CPR quality in the prehospital patient care record. Fourth, our public health measures may change according to the COVID-19 pandemic severity, and it is challenging to evaluate the real impact of the pandemic on pediatric OHCA. Such limitations may cause some bias in analyzing the pediatric OHCA before and during the pandemic. Further studies prospectively collecting data on pediatric OHCA would be required.

## Conclusion

Out-of-hospital cardiac arrest is relatively uncommon in children and usually has poor outcomes. We found a significantly shorter survival duration of pediatric patients with OHCA during the COVID-19 pandemic than in the pre-pandemic period. The COVID-19 pandemic may change the nature of pediatric ED visits and affect the factors related to STHD in pediatric patients with OHCA. A larger study is needed to examine the comprehensive impacts of the COVID-19 pandemic on the outcome of pediatric OHCA and provide information not only to improve pre-and in-ED preparation and management but also to prevent the occurrence of OHCA in children.

## Data Availability Statement

The original contributions presented in the study are included in the article/supplementary material, further inquiries can be directed to the corresponding authors.

## Ethics Statement

The studies involving human participants were reviewed and approved by the China Medical University Hospital. Written informed consent from the participants’ legal guardian/next of kin was not required to participate in this study in accordance with the national legislation and the institutional requirements.

## Author Contributions

C-YC and E-PL reviewed the medical records, analyzed and interpreted the data, and drafted the manuscript. Y-JC and W-CY analyzed and interpreted the data. H-PW and M-JL designed and oversaw the study, interpreted the data, and revised the manuscript. All authors have read and approved the final manuscript for publication.

## Conflict of Interest

The authors declare that the research was conducted in the absence of any commercial or financial relationships that could be construed as a potential conflict of interest.

## Publisher’s Note

All claims expressed in this article are solely those of the authors and do not necessarily represent those of their affiliated organizations, or those of the publisher, the editors and the reviewers. Any product that may be evaluated in this article, or claim that may be made by its manufacturer, is not guaranteed or endorsed by the publisher.
